# Predicting aggressive disease and poor outcome in endometrial cancer using preoperative [^18^F]FDG PET primary tumor radiomics

**DOI:** 10.1007/s00259-025-07335-7

**Published:** 2025-06-11

**Authors:** Kristine Eldevik Fasmer, Ankush Gulati, Sunniva Lindås, Camilla Krakstad, Ingfrid Salvesen Haldorsen

**Affiliations:** 1 Mohn Medical Imaging and Visualization Centre, Department of Radiology, Haukeland University Hospital, Bergen, Norway; 2 Department of Clinical Medicine, University of Bergen, Bergen, Norway; 3 Centre for Cancer Biomarkers, Department of Clinical Science, University of Bergen, Bergen, Norway; 4 Department of Obstetrics and Gynecology, Haukeland University Hospital, Bergen, Norway

**Keywords:** Endometrial cancer, Positron emission tomography computed tomography, Radiomics, Fluorodeoxyglucose F18, Prognosis

## Abstract

**Purpose:**

To develop a [^18^F]fluorodeoxyglucose ([^18^F]FDG) positron emission tomography (PET) primary tumor radiomic model for predicting disease-specific survival (DSS), and compare it with conventional PET markers in a large endometrial cancer cohort.

**Methods:**

Radiomic features were extracted from preoperative [^18^F]FDG PET scans of 489 endometrial cancer patients using a standardized uptake value (SUV) threshold > 2.5 to define primary metabolic tumor volumes (MTVs). A second reader extracted features in 154/489 patients, in which intraclass correlation coefficients (ICCs) were calculated. Radiomic features with ICCs > 0.75 were retained and ComBat harmonization was applied to reduce scanner/protocol effects on the extracted features. Patients were divided into training (*n* = 343) and test (*n* = 146) sets. A radiomic DSS score (R_dss_) was developed in the training set using least absolute shrinkage and selection operator (LASSO) Cox regression. A combined model (C_dss_), incorporating R_dss_, PET positive lymph nodes (LN_PET_) and preoperative histology risk was constructed using multivariable Cox hazard analyses. Prediction performances were assessed by comparing areas under time-dependent receiver operating characteristic curves (tdROCs AUCs) for R_dss_, C_dss_, and conventional PET markers: SUV_max_, SUV_mean_, MTV, tumor lesion glycolysis (TLG) and LN_PET_.

**Results:**

In the test set, AUCs for 2- and 5-year DSS were higher for R_dss_ (0.855, 0.720) compared to SUV_max_ (0.548, 0.572) and SUV_mean_ (0.549, 0.554) (*p* ≤ 0.04 for all), while similar to MTV (0.863, 0.696), TLG (0.814, 0.672) and LN_PET_ (0.802, 0.626) (*p* ≥ 0.12 for all). C_dss_ predicted 2-year DSS with AUC of 0.909 in the test set, outperforming all conventional imaging markers (*p* ≤ 0.04 for all) except MTV (*p* = 0.29). For 5-year DSS, C_dss_ (AUC: 0.817) outperformed all conventional imaging markers, including MTV (AUC ≤ 0.696, *p* ≤ 0.05, for all).

**Conclusion:**

R_dss_ predicts short-term survival with high accuracy, outperforming tumor SUV_max/mean_, but not MTV, TLG and LN_PET_. The combined C_dss_ model yields high accuracy for predicting both short- and long-term survival, outperforming all conventional PET imaging markers.

**Supplementary Information:**

The online version contains supplementary material available at 10.1007/s00259-025-07335-7.

## Introduction

The number of endometrial cancer cases is steadily increasing in medium- to high-income countries, with 420,242 new cases diagnosed worldwide annually, including 817 in Norway (numbers from 2022 [1, 2]). This rising incidence is primarily attributed to increasing obesity rates and longer life expectancies. Precise diagnoses and effective, personalized treatment options remain significant clinical challenges in current endometrial cancer patient care.

Preoperative histological classification categorizes endometrial cancer patients into low-risk (endometrioid endometrial carcinoma (EEC) grade 1–2) and high-risk (EEC grade 3–4 and non-endometrioid endometrial carcinoma (NEEC)) groups [3]. Additionally, preoperative magnetic resonance imaging (MRI) and positron emission/computed tomography imaging (PET/CT) are commonly used to assess disease extent and tailor primary and adjuvant treatments [4–7]. While pelvic MRI is highly specific for assessing locoregional tumor extent [6, 8], [^18^F]fluorodeoxyglucose ([^18^F]FDG) PET/CT is superior for evaluating lymphadenopathy and distant metastases in endometrial cancer [9, 10].

Over the last decades, radiomics has evolved as an imaging analysis technique aimed at providing tumor characteristics at a mesoscopic level allowing in vivo phenotyping of tumors. This is achieved by recognizing image patterns that potentially reflect underlying tumor pathophysiology, e.g. necrosis, proliferation, histologic features, or genetic alterations. These radiomic patterns can potentially identify aggressive and treatment-resistant tumor profiles, and predict patient outcomes [11–13].

In endometrial cancer, several studies have explored primary tumor radiomics in preoperative MRI for predicting aggressive disease and poor outcome [14–20]. While standard MRI images are inherently ‘anatomical’, [^18^F]FDG PET images provide ‘metabolic’ information on increased tissue glycolysis. Endometrial cancers are known to exhibit increased [^18^F]FDG uptake, and previous studies have demonstrated medium-to-high accuracies in predicting aggressive disease and poor outcomes using routinely reported quantitative metabolic markers for primary- and metastatic tumors [6, 7, 21, 22]. However, the prognostic potential of more advanced metabolic radiomic tumor profiling has previously been minimally explored [23–25]. In the present study, we are investigating preoperative primary tumor [^18^F]FDG PET radiomics in a large, population-based cohort of endometrial cancer patients. The main aim is to develop PET radiomic tumor models for predicting aggressive disease and patient outcomes, and to compare their predictive performance with routinely reported conventional PET imaging markers.

## Material and methods

### Patient cohort

**Fig. 1:**
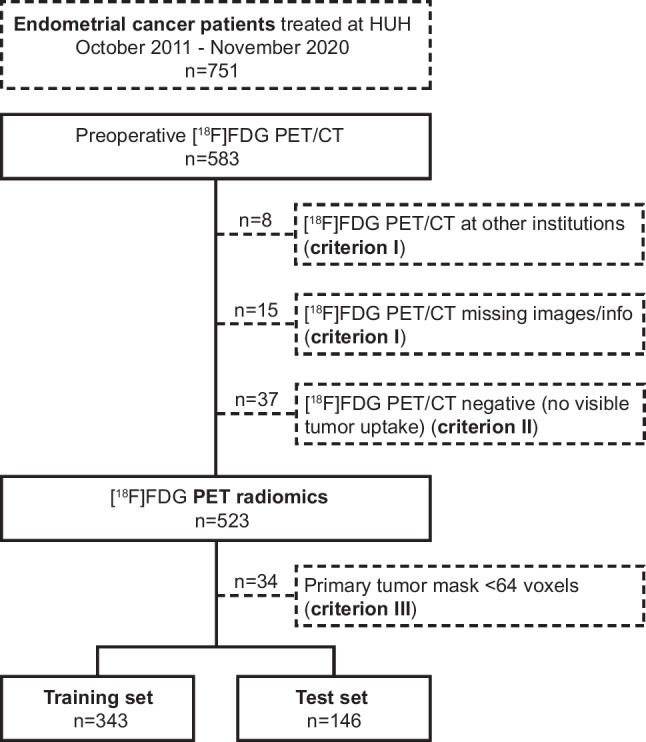
**Study flowchart**. From October 2011 to November 2020, 751 patients were treated for endometrial cancer at Haukeland University Hospital (HUH, Bergen, Norway). Among these, 583 patients underwent pretreatment [^18^F]fluoro-deoxy-glucose positron emission/computed tomography ([^18^F]FDG PET/CT). A total of 60 out of 583 patients were excluded due to missing images/series (*n* = 15, inclusion criterion I), PET/CT performed at other institutions (*n* = 8, inclusion criterion I), or no visible tumor tracer uptake (*n* = 37, inclusion criterion II). Additionally, 34 patients were excluded during radiomic feature extraction, due to having fewer than 64 voxels in the segmented primary tumor masks (inclusion criterion III). The remaining 489 patients were included for further analyses and divided in a radiomic model training set (*n* = 343), and a test set (*n* = 146)

This retrospective study on prospectively collected data was approved by the Regional Committee for Medical Research Ethics (2015/2333/REK vest) with written informed consent obtained at primary diagnosis from all enrolled patients. Patients admitted to Haukeland University Hospital from October 2011 to November 2020 with histologically verified endometrial cancer and preoperative [^18^F]FDG PET/CT scans available in the hospital’s clinical archiving system (PACS) were enrolled (*n* = 583) (Fig. 1). The inclusion criteria were as follows: (I) complete, preoperative [^18^F]FDG PET/CT performed with standardized protocols at Haukeland University Hospital, (II) visible primary tumor tracer uptake, and (III) primary tumor volume (segmented tumor mask) incorporating > 64 image voxels (Fig. 1). In total, 489 patients met the inclusion criteria and were allocated in a 70/30 split to a radiomic model training set (*n* = 343) and testing set (*n* = 146). The split was performed by balancing disease-specific survival (DSS) in the training and test sets (Table 1).

**Table 1:** **Study cohort overview**. Clinical and pathological characteristics of the 489 endometrial cancer patients in the study cohort (treated October 2011-November 2020). The patients underwent preoperative [^18^F]FDG PET/CT on two different scanners (Biograph TruePoint and Biograph Vision, both Siemens). The study cohort is divided in a radiomic model training set (n = 343) and testing set (n = 146), and clinicopathological patient characteristics are compared between the sets (p*)

	Study cohort (*n* = 489)	Training set (*n* = 343)	Test set (*n* = 146)	p*
Age (years), median [IQR]	68 [62, 74]	69 [62, 74]	67 [60, 74]	.55
BMI^a^ (kg/m^2^), median [IQR]	28 [25, 33]	28 [25, 32]	29 [26, 34]	**.02**
Menopausal status^a^, n (%)				.85
pre/perimenopausal	35 (7)	24 (7)	11 (8)	
postmenopausal	453 (93)	319 (93)	134 (92)	
Preoperative histology^a^				.25
low-risk (EEC grade 1–2)	315 (65)	215 (64)	100 (69)	
high-risk (EEC grade 3/NEEC)	166 (35)	122 (36)	44 (31)	
PET/CT scanner, n (%)				.39
Biograph TruePoint	348 (71)	240 (70)	108 (74)	
Biograph Vision	141 (29)	103 (30)	38 (26)	
Primary treatment, n (%)				.16
hysterectomy	474 (97)	335 (98)	139 (95)	
curettage/palliative surgery	15 (3)	8 (2)	7 (5)	
Lymphadenectomy, n (%)				.23
yes	292 (60)	211 (62)	81 (55)	
no	197 (40)	132 (38)	65 (45)	
Lymph node metastasis^b^, n (%)				.86
no	247 (85)	179 (85)	68 (84)	
yes	45 (15)	32 (15)	13 (16)	
Histological subtype and grade^a^^,b^, n (%)				.12
EEC grade 1	193 (40)	139 (41)	54 (37)	
EEC grade 2	118 (24)	74 (22)	44 (30)	
EEC grade 3	61 (13)	40 (12)	21 (14)	
NEEC	112 (23)	85 (25)	27 (19)	
FIGO 2009 stage^b^, n (%)				.36
I	362 (74)	257 (75)	105 (72)	
II	45 (9)	30 (9)	15 (10)	
III	60 (12)	44 (13)	16 (11)	
IV	22 (5)	12 (3)	10 (7)	
Adjuvant treatment, n (%)				.92
no	291 (60)	205 (60)	86 (59)	
yes^c^	198 (40)	138 (40)	60 (41)	
Disease specific survival				.89
alive/dead from other causes	414 (85)	291 (85)	123 (84)	
dead from disease	75 (15)	52 (15)	23 (16)	

*BMI* body mass index, *EEC* endometrioid endometrial carcinoma, *FIGO* International Federation of Gynecology and Obstetrics, *IQR* interquartile range, *NEEC* non-endometrioid endometrial carcinoma, *[*^*18*^*F]FDG PET/CT*, positron emission tomography combined with computed tomography using [^18^F]fluorodeoxyglucose

^a^Number (n) of patients with variables missing/not assessed in the study cohort: BMI (1), menopausal status (1), preoperative histology (8), surgicopathological histology or grade (5)

^b^Surgicopathological assessments

^c^Adjuvant treatment was chemotherapy (*n* = 176), radiation therapy (*n* = 4), brachytherapy (*n* = 3), hormonal treatment (*n* = 4), chemoradiation (*n* = 7) and other treatment (*n* = 4)

^*^Mann–Whitney exact U-test for continuous variables, and Fisher’s exact test for categorical variables, comparing clinicopathological patient characteristics in the training, and in the test set (*p* < 0.05 marked in bold)

### Patient information and clinical data

Clinical and histopathological patient data including age, menopausal status, histologic tumor type and grade, primary treatment and follow-up information were collected from medical records. Preoperative endometrial biopsies were histologically classified as low-risk (EEC grade 1–2) or high-risk (EEC grade 3/NEEC). All patients were surgically staged according to the International Federation of Gynecology and Obstetrics (FIGO) 2009 criteria. Primary treatment involved hysterectomy with bilateral salpingo-oophorectomy. Lymphadenectomy was performed in selected patients, based on preoperative risk assessments incorporating information on preoperative histology and radiological findings. Patients assessed as high-risk based on final surgicopathologic stage received adjuvant treatment, standardly chemotherapy (carboplatin and paclitaxel) in line with national treatment guidelines [26]. Disease-specific survival (DSS) was defined as the time from primary treatment to death due to endometrial cancer or to the last follow-up.

### PET/CT imaging protocols

Patient preparation, image acquisition, and image reconstruction were in accordance with the EANM (European Association of Nuclear Medicine) guidelines version 2.0 [27]. Patients were instructed to fast for 6 h, and blood glucose levels were controlled to be below 180 mg/dL prior to scanning. After intravenous injection of [^18^F]FDG, the patients rested for approximately 60 min prior to scanning. PET/CT images were acquired from the skull base to mid-thigh and all PET images were corrected for attenuation and scatter using CT data. Prior to 2018, patients were scanned using Siemens Biograph TruePoint (348/489 patients), and after a scanner upgrade in 2018, patients were scanned using Siemens Biograph Vision (141/489 patients). The acquisition and reconstruction settings for both scanners are detailed in Online Resource 1.

### PET/CT image review and tumor segmentation

All PET/CT images were reviewed using Segami Oasis v1.9 (https://www.segamicorp.com/) by a nuclear medicine physician with more than six years of experience (Reader 1), who was blinded to patients’ clinical, pathological, and outcome data, as well as other imaging findings. Increased [^18^F]FDG uptake in the primary tumor, lymph nodes (LN_PET_), and at distant sites indicating metastases, were recorded in PET registration forms. Standardized uptake value (SUV) was defined as (tissue radioactivity concentration [Bq/mL]) × (body weight [g])/(injected radioactivity [Bq]). The outer primary tumor border was delineated by Reader 1 using a freely available software (LIFEx v7.4.0, https://www.lifexsoft.org/) [28], and a lower SUV threshold of 2.5 was applied to define the primary metabolic tumor volume (MTV).

A separate reviewer (Reader 2, a medical student trained by Reader 1), independently segmented MTVs for 154/489 patients, using the > 2.5 SUV threshold. Data from reader 2 were utilized to assess interobserver reliability between the two readers, for the extracted primary tumor radiomic features.

### Radiomic feature extraction and reduction

**Fig. 2:**
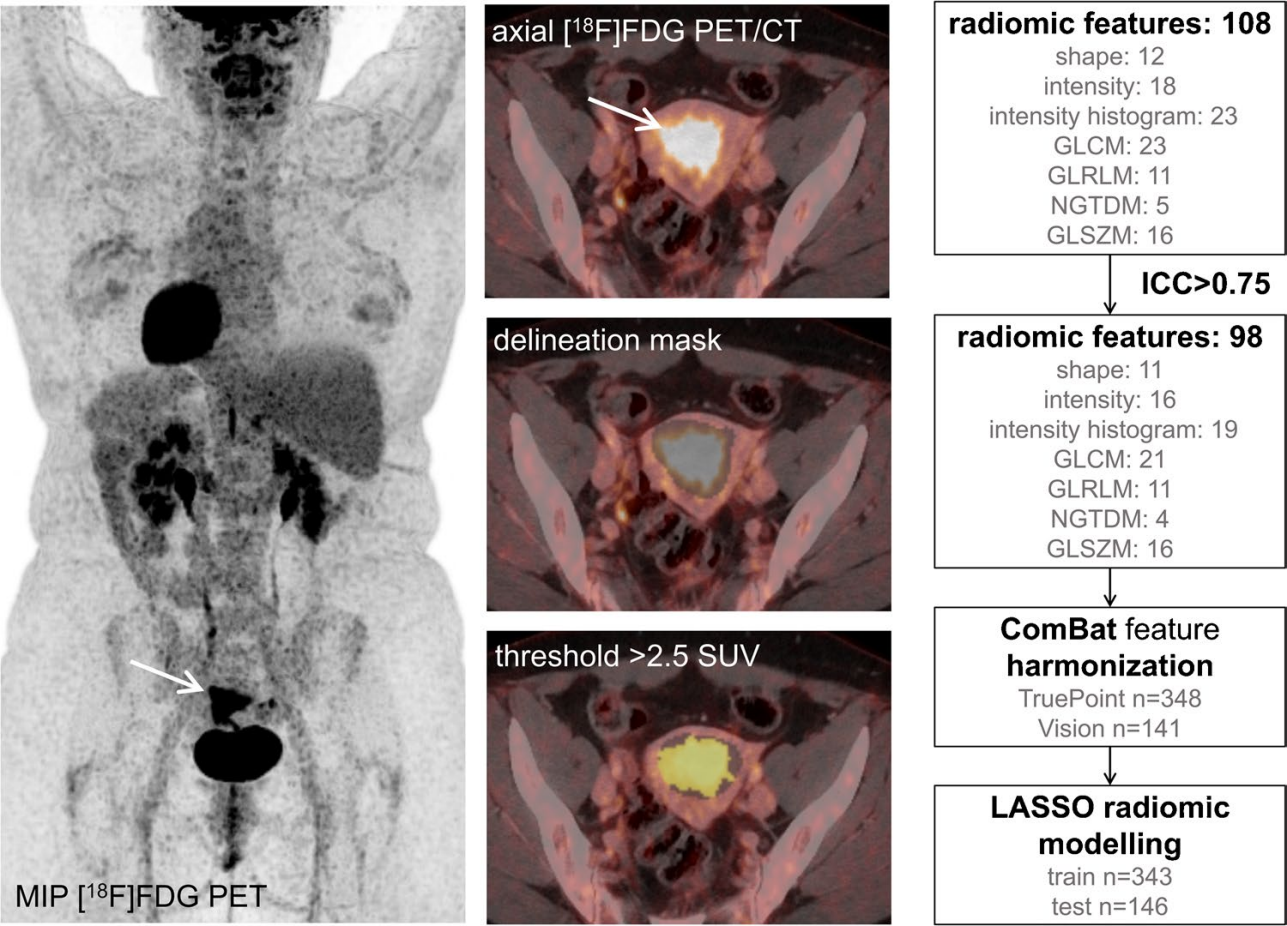
**Tumor segmentation, feature selection and radiomic modelling**. [^18^F]FDG PET MIP (*left*), and axial PET/CT slices (*middle*) showing a primary endometrial tumor (arrow) in a patient with grade 1 endometrioid endometrial carcinoma and International Federation of Gynecology and Obstetrics (FIGO) stage IA. The primary tumor was delineated using a masking tool in every slice covering the entire tumor. Metabolic tumor volume was defined by applying a SUV threshold of > 2.5, of which 108 radiomic features were extracted. Radiomic features with ICCs > 0.75 were retained for further analyses. To account for interscanner/protocol effects, the 98 included radiomic features were ComBat harmonized, before radiomic models predicting aggressive disease were developed (*n* = 343) and subsequently tested (*n* = 146). [^18^F]FDG PET/CT, [^18^F]fluorodeoxyglucose positron emission/computed tomography; GLCM, grey-level co-ocurrence matrix; GLRLM, grey-level run-length matrix; GLSZM, grey-level size-zone matrix; ICC, intraclass correlation coefficient; LASSO, least absolute shrinkage and selection operator; MIP, maximum intensity projection; NGTDM, neighboring grey-tone difference matrix; SUV, standardized uptake value

PET radiomic features were extracted in LIFEx, using minimum/maximum intensity bounds set to 0/40 SUV, intensity discretization to 128 grey levels (corresponding to a bin size of 0.32 SUV), and 3D dimension processing. In total, 108 IBSI-compliant [29] (https://theibsi.github.io/) PET radiomic features were extracted from the segmented MTVs (Fig. 2).

Only radiomic features with high interobserver agreement (intraclass correlation coefficient (ICC) > 0.75) were retained for further analyses (Online Resource 2). Subsequently, post hoc ComBat harmonization [30] was performed using an online tool (https://forlhac.shinyapps.io/Shiny_ComBat/), in order to reduce scanner/protocol effects on the extracted radiomic features (e.g., different voxel sizes). An extensive overview of the radiomic feature distribution before and after ComBat harmonization is provided in Online Resource 3.

### Prediction modelling and model performance assessments

Following feature extraction and reduction, the least absolute shrinkage and selection operator (LASSO) Cox regression method [31] was applied to the training set, to select features with high predictive power of DSS. The regularization parameter (λ) was optimized by tenfold cross-validation (Online Resource 4). Selected features were further linearly combined with respective model coefficients (Online Resource 5), to compute radiomic scores for each patient (R_dss_), in both the training (*n* = 343) and test sets (*n* = 146) (Fig. 2). The performance of R_dss_ was evaluated in both sets for the prediction of DSS, as well as for the prediction of surgicopathological lymph node metastases (LNM), advanced FIGO stage (stage III-IV), and high-risk histology (EEC grade 3/NEEC in surgical specimen). The performance of R_dss_ was further compared to that of well-established PET features: SUV_max_, SUV_mean_, MTV, tumor lesion glycolysis (TLG = MTV × SUV_mean_), and LN_PET_.

Univariable and multivariable Cox proportional hazard analyses were used to determine DSS hazard ratios (HRs) for the R_dss_, the conventional imaging findings SUV_max_, MTV, and LN_PET_, and the preoperative histology risk in the training set. Variables with significant HRs in multivariable analyses were retained, and the Cox model coefficients were used to compute a combined score for each patient (C_dss_), both in the training and test sets. The performance of C_dss_ was evaluated and compared with R_dss_ for predicting DSS, surgicopathological LNM, FIGO III-IV, and high-risk histology.

In addition to the DSS models R_dss_ and C_dss_, specific LASSO radiomic models were developed for predicting LNM (R_lnm_), FIGO stage III-IV (R_figo_) and surgicopathological high-risk histology (R_hist_). The models were developed in the training set (Online Resources 4–5), and compared with R_dss_ and C_dss_ in the training and test set.

### Statistical analyses

The statistical analyses were performed in R version 4.4.1 (https://www.r-project.org/) and STATA version 18.0 (StataCorp LLC, College Station, TX, USA). For comparisons of clinicopathological patient characteristics across the training and test sets, Mann–Whitney exact U-test was applied for continuous variables, while Fisher’s exact test was applied for categorical variables. To assess the interobserver reliability of the radiomic features, ICCs were derived using a two-way random model (individual absolute agreement).

In R, the “caret” package was used for computing a balanced cohort split with regard to DSS, the “glmnet” package was used for LASSO regression, the “timeROC” package was used to compute time-dependent receiver operating characteristics (tdROC) curves for the prediction of DSS 1- to 5-year after primary treatment, and the “pROC” package was used to compute ROC for prediction of LNM, FIGO III-IV, and high-risk histology. Area under the tdROC/ROC curves (AUC) were compared using DeLong’s test.

Univariable and multivariable Cox proportional hazard analyses were performed in STATA. Variables with significant HRs in multivariable analyses were used to compute the combined score C_dss_. All variables in C_dss_ satisfied the assumption of proportional hazards (the Schoenfeld test of residuals and graphical diagnostics).

Optimal cut-offs for the R_dss_ and C_dss_ models were identified by Youden Index from the 5-year DSS tdROCs in the training set, and the patients were subsequently divided into high- and low R_dss_/C_dss_ score groups. Differences in DSS between the groups were explored by Kaplan–Meier analyses with log-rank tests.

p-values of less than 0.05 were considered to represent statistically significant findings.

## Results

### Clinical findings, histopathology and patient survival

Haukeland University Hospital (HUH) is a full-scale gynecologic oncology center, serving approximately 10% of the Norwegian population. In addition, the center receives high-risk/advanced stage patients from neighboring Hospital Trusts. Whole-body [^18^F]FDG PET/CT was gradually implemented in the clinical diagnostics starting in 2011, reaching over 85% of the endometrial cancer patients by 2019 [32]. From October 2011 to November 2020, a total of 751 endometrial cancer patients were diagnosed and treated at the center, of which 78% (583/751) had preoperative [^18^F]FDG PET/CT available in PACS. Out of the patients with preoperative PET/CT, 489/583 patients were included in the study cohort (23 patients were excluded due to inclusion criterion (I), 37 due to (II) and 34 due to (III)) (Fig. 1).

In the study cohort, the observed time interval from PET/CT to primary treatment had a median (interquartile range (IQR)) of 17 (9–27) days. The median (IQR) age was 68 (62–74), and 97% (474/489) of the patients underwent hysterectomy as primary treatment (Table 1). Lymphadenectomy was performed in 60% (292/489) of the patients and lymph node metastases were surgically confirmed in 15% (45/292) of these patients. By surgicopathological assessments, 74% (362/489) of patients were staged as FIGO I, which corresponds well with nationally reported cancer staging numbers: 71% of endometrial cancer patients in Norway being diagnosed with localized disease [33]. Adjuvant treatment was given to 40% (198/489) of the patients; chemotherapy in 176/198 patients, chemoradiation in 7/198, external radiation in 4/198, brachytherapy in 3/198, hormonal treatment in 4/198, and other treatment in 4/198. Median (IQR) [range] follow-up time for survivors was 60 (45, 70) [3, 101] months.

For model training and testing, the patients were divided into two subcohorts, 70% (343/489) of the cases were used for model training, while 30% (146/489) were reserved for model evaluation. The clinicopathological patient characteristics were overall similar between the training- and test cohorts (Table 1).

### Tumor segmentation and radiomic feature extraction

A total of 108 IBSI-compliant primary tumor radiomic features were extracted by Reader 1 for the 489 patients in the study cohort. A comprehensive list of radiomic features is provided in Online Resource 2. The interobserver reliability for each of the 108 features was assessed by calculating ICCs between Reader 1 and 2 for the 154 overlapping patient cases. The ICC for MTV was excellent between the two readers (MTV ICC: 0.97). A total of ten features with ICCs ≤ 0.75 were excluded, leaving 98 radiomic features for radiomic modelling (Online Resource 2).

### Radiomic model for prediction of disease-specific survival

Radiomic LASSO Cox modelling was performed utilizing the 98 ComBat-harmonized radiomic features (Online Resource 3) for the prediction of DSS. A radiomic score/signature was constructed by combining LASSO-selected radiomic features with corresponding model coefficients (Online Resource 5):

$${R}_{dss} = \left(0.000011\times SurfaceAre{a}_{Morph}\right)+\left(0.00093\times Max3DDiamete{r}_{Morph}\right)+\left(0.00099\times MaxHistGradien{t}_{IntHist}\right)+\left(0.79025\times NormInvDifferenc{e}_{GLCM}\right)$$

**Fig. 3:**
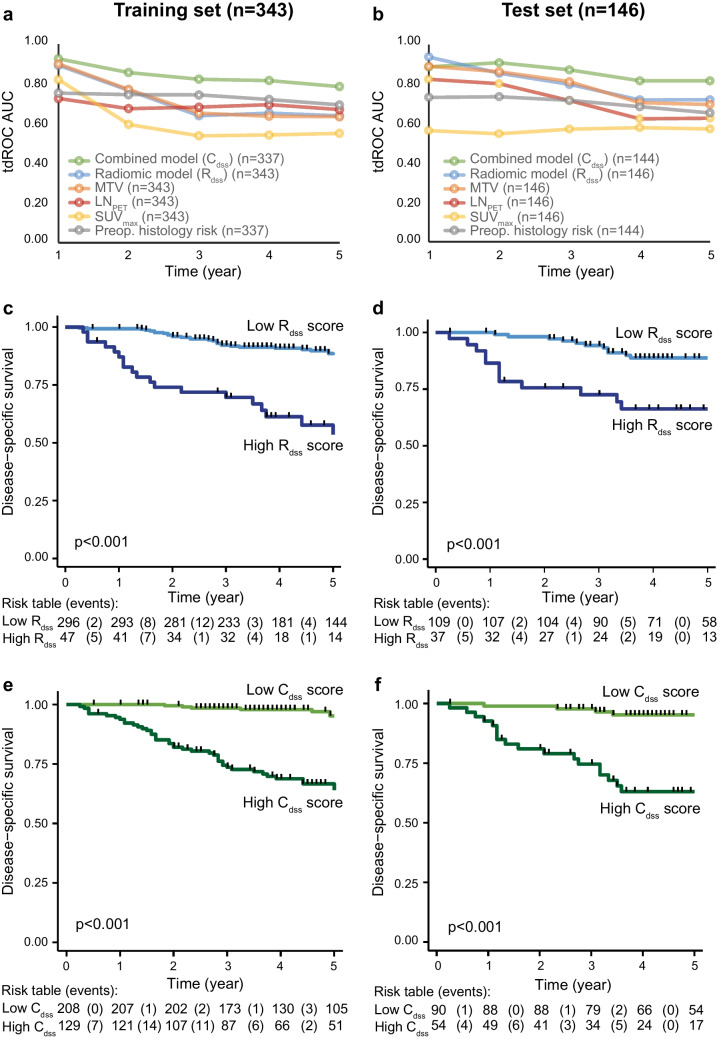
**Radiomic- and combined models for predicting survival in endometrial cancer.** AUCs tdROCs for predicting 1- to 5-year DSS for the radiomic model (R_dss_), the combined model (C_dss_), the conventional imaging markers SUV_max_, MTV, LN_PET_, and preoperative histology risk (low-risk: endometrioid endometrial carcinoma (EEC) grade 1–2; high-risk: EEC grade 3/non-endometrioid endometrial carcinoma), in the traning (**a**) and test (**b**) sets. Kaplan–Meier curves depicting DSS in patients with low- versus high scores of R_dss_ (**c**, **d**) and C_dss_ (**e**, **f**) in the training- (**c**, **e**) and test (**d**, **f**) sets, respectively. Optimal cutoff values for R_dss_ and C_dss_ were identified from the 5-year DSS tdROC curves in the training set, using Youden Index. AUC, area under curve; DSS, disease-specific survival; LN_PET_, visually suspected lymph node metastases from PET reading; MTV, metabolic tumor volume; SUV_max_, maximum primary tumor standardized uptake value; tdROC, time-dependent receiver operating characteristic curve

R_dss_ predicted 2-year DSS with tdROCs AUCs of 0.766/0.855 in training/test, and 5-year DSS with tdROCs AUCs of 0.639/0.720 in the training/test (Table 2., Fig. 3a, b). In the test, both 2- and 5-year AUCs of R_dss_ were significantly higher than those of tumor SUV_max_ and SUV_mean_ (*p* ≤ 0.04 for all). However, when comparing R_dss_ with MTV, TLG, and LN_PET_, there were no significant AUCs differences in the test set, neither for 2- nor 5-year DSS (*p* ≥ 0.12 for all) (Table 2.).

**Table 2.** **Radiomic- and combined models compared with conventional PET markers for prediction of aggressive disease and survival**. Area under curve (AUC) of time-dependent (td) receiver operating curves (ROC) for the radiomic model (R_dss_), the combined model (C_dss_), and the conventional PET imaging markers (SUV_max_, SUV_mean_, MTV, TLG, and LN_PET_) for predicting disease-specific survival (DSS) at 2 and 5 years after diagnosis, and AUCs ROCs for predicting surgicopathological lymph node metastases, FIGO stage III-IV, and high-risk histology. Pairwise AUC comparisons of C_dss_ versus R_dss_ and conventional PET markers (p^a^), and of R_dss_ versus conventional PET markers (p^b^) are given. All analyses are performed both in the training- (n = 343) and test (n = 146) sets

	C_dss_^e^	R_dss_	SUV_max_	SUV_mean_	MTV	TLG	LN_PET_
Disease-specific survival (AUC tdROC@2y)							
training (*p*^a^/*p*^b^), *n* = 343	.857 (-/-)	.766 (*/-)	.596 (**/**)	.575 (**/**)	.772 (*/ns)	.764 (*/ns)	.675 (**/ns)
test (*p*^a^/*p*^b^), *n* = 146	.909 (-/-)	.855 (ns/-)	.548 (**/**)	.549 (**/**)	.863 (ns/ns)	.814 (*/ns)	.802 (*/ns)
Disease-specific survival (AUC tdROC@5y)							
training (*p*^a^/*p*^b^), *n* = 343	.786 (-/-)	.639 (**/-)	.551 (**/ns)	.573 (**/ns)	.634 (**/ns)	.635 (**/ns)	.670 (**/ns)
test (*p*^a^/*p*^b^), *n* = 146	.817 (-/-)	.720 (ns/-)	.572 (**/*)	.554 (**/*)	.696 (*/ns)	.672 (*/ns)	.626 (**/ns)
Lymph node metastases^c^ (AUC ROC)							
training (*p*^a^/*p*^b^), *n* = 211	.820 (-/-)	.686 (**/-)	.595 (**/ns)	.597 (**/ns)	.701 (**/ns)	.690 (**/ns)	.708 (**/ns)
test (*p*^a^/*p*^b^), *n* = 81	.800 (-/-)	.636 (ns/-)	.494 (**/ns)	.517 (**/ns)	.651 (ns/ns)	.626 (ns/ns)	.818 (ns/ns)
FIGO III-IV (AUC ROC)							
training (*p*^a^/*p*^b^), *n* = 343	.857 (-/-)	.740 (**/-)	.656 (**/*)	.680 (**/ns)	.773 (**/*)	.775 (**/ns)	.713 (**/ns)
test (*p*^a^/*p*^b^), *n* = 146	.805 (-/-)	.739 (ns/-)	.620 (**/ns)	.610 (**/ns)	.743 (ns/ns)	.729 (ns/ns)	.770 (ns/ns)
High-risk histology^d^ (AUC ROC)							
training (*p*^a^/*p*^b^), *n* = 338	.886 (-/-)	.612 (**/-)	.594 (**/ns)	.621 (**/ns)	.672 (**/**)	.677 (**/**)	.545 (**/*)
test (*p*^a^/*p*^b^), *n* = 146	.869 (-/-)	.686 (**/-)	.602 (**/ns)	.598 (**/ns)	.677 (**/ns)	.671 (**/ns)	.590 (**/ns)

*FIGO* International Federation of Gynecology and Obstetrics, *LN*_*PET*_ visually suspected lymph node metastases from PET reading, *MTV* metabolic primary tumor volume, *PET* positron emission tomography, *SUV*_*mean*_ mean primary tumor standardized uptake value, *SUV*_*max*_ maximum primary tumor standardized uptake value, *TLG* tumor lesion glycolysis (= MTV × SUV_mean_), *y* year

^a^*p*-value (deLongs test) for pairwise area under curve comparisons between C_dss_ versus R_dss_ and the conventional PET imaging markers. *p* < 0.05 denoted (*), *p* < 0.01 denoted (**), *p* ≥ 0.05 denoted ns (not significant)

^b^*p*-value (deLongs test) for pairwise area under curve comparisons between R_dss_ versus the conventional PET imaging markers. *p* < 0.05 denoted (*), *p* < 0.01 denoted (**), *p* ≥ 0.05 denoted ns (not significant)

^c^Lymphadenectomy in *n* = 292 patients

^d^Endometrioid grade 3 or non-endometrioid endometrial carcinoma. Grade missing for five patients

^e^Preoperative histology missing for eight patients. AUCs of C_dss_ and p-value comparisons with C_dss_ are therefore reported for in total *n* = 481 patients for disease-specific survival and FIGO, *n* = 207 patients for lymph node metastases, *n* = 477 patients for high-risk histology

The optimal cut-off for R_dss_, based on 5-year DSS tdROC in the training set, was 1.026. In Kaplan–Meier analyses, patients with higher R_dss_ scores (above the cut-off), had significantly poorer DSS than patients with lower R_dss_ scores (below the cut-off), both in the training set (Fig. 3c, *p* < 0.001) and in the test set (Fig. 3d, *p* < 0.001).

### Combined model for prediction of disease-specific survival

In univariable Cox analyses, R_dss_ (HR: 5.3), MTV (HR: 1.003), LN_PET_ (HR: 6.7) and preoperative histology risk (HR: 5.6) were all significant predictors of DSS (*p* ≤ 0.004 for all), while SUV_max_ was not (*p* = 0.22) (Table 3). In multivariable Cox analyses, only R_dss_, LN_PET_ and preoperative histology risk remained significant predictors of DSS (*p* ≤ 0.002 for all) (Table 3). Using the resulting Cox model coefficients for these three predictors, a combined score (C_dss_) was constructed:

**Table 3:** **Cox regression analyses for the radiomic tumor score, conventional PET markers, and preoperative histology risk**. Univariable and multivariable Cox regression analyses of the radiomic score (R_dss_), conventional PET markers (SUV_max_, MTV, LN_PET_) and preoperative histology riska for prediction of disease-specific survival (DSS) in the model training set (n = 343). The multivariable analyses incorporated all variables with significant hazard ratios (HRs) in univariable analyses. Variables that remained significant in multivariable analyses were used to construct a combined DSS score using the resulting Cox model coefficients (C_dss_)

	Univariable analyses		Multivariable analyses		Cox combined model (C_dss_)		
Preoperative variables	HR (95% CI)	p*	HR (95% CI)	p*	HR (95% CI)	coefficient	p*
R_dss_ (*n* = 343)	5.3 (2.7, 10.6)	< **.001**	12.5 (2.6, 60.5)	**.002**	3.3 (1.4, 7.9)	1.205	**.006**
SUV_max_ (*n* = 343)	1.02 (0.99, 1.06)	.22	-	-	-	-	-
MTV (*n* = 343)	1.003 (1.001, 1.005)	**.004**	1.00 (0.99, 1.00)	.16	-	-	-
LN_PET_ (*n* = 343)	6.7 (3.8, 11.8)	< **.001**	4.6 (2.6, 8.2)	< **.001**	4.6 (2.6, 8.1)	1.518	< **.001**
Preoperative histology risk^a^ (*n* = 337)	5.6 (3.0, 10.6)	< **.001**	4.0 (2.1, 7.7)	< **.001**	3.9 (2.0, 7.6)	1.368	< **.001**

*CI* confidence interval, *LN*_*PET*_ visually suspected lymph node metastases from PET reading, *MTV* metabolic primary tumor volume, *PET* positron emission tomography, *SUV*_*max*_ maximum primary tumor standardized uptake value

^a^Low-risk: endometrioid endometrial carcinoma (EEC) grade 1–2; high-risk: EEC grade 3 and non-endometrioid endometrial carcinoma

^*^Hazard ratios/model coefficients with *p* < 0.05 given in bold

$${C}_{dss} = \left(1.205\times {R}_{dss}\right)+\left(1.518\times {LN}_{PET}\right)+\left(1.368\times Preop.Hist.Risk\right)$$

C_dss_ predicted 2-year DSS with tdROCs AUCs of 0.857/0.909 in training/test sets, outperforming all conventional imaging markers (*p* ≤ 0.04 for all), except MTV (test set AUC: 0.863, *p* = 0.29) (Table 2.). Moreover, C_dss_ predicted 5-year DSS with tdROCs AUCs of 0.786/0.817 in the training/test sets (Table 2.), outperforming all conventional imaging markers, including MTV (*p* < 0.05 for all). Compared to R_dss_, C_dss_ yielded significantly higher 2- and 5-year AUCs in the training set (*p* ≤ 0.01 for all), while similar AUCs in the test set (*p* > 0.05 for all).

In Fig. 3a, b, AUCs tdROCs are provided for all timepoints (1- to 5-year DSS) for C_dss_, R_dss_, the conventional imaging markers SUV_max_, MTV, LN_PET_, and preoperative histology risk. C_dss_ demonstrated overall the highest AUCs for 2- to 5- year DSS, both in the training set (C_dss_ AUCs: 0.786–0.857, Fig. 3a) and in the test set (C_dss_ AUCs: 0.817–0.909, Fig. 3b).

The optimal cut-off for C_dss_, based on 5-year DSS tdROC in the training set, was 3.601. Kaplan–Meier analyses demonstrated excellent separation in the DSS curves for patients with higher C_dss_ scores (above the cut-off), compared to patients with lower C_dss_ scores (below the cut-off), both in the training set (Fig. 3e, *p* < 0.001) and in the test set (Fig. 3f, *p* < 0.001).

### Radiomic- and combined models for prediction of lymph node metastases, advanced stage and high-risk histology

Applying the radiomic model R_dss_ for predicting aggressive disease markers yielded ROC AUCs of 0.686/0.636 for LNM, 0.740/0.739 for FIGO III-IV, and 0.612/0.686 for high-risk histology in the training/test sets (Table 2.). Comparing the prediction performance of R_dss_ with conventional imaging markers in the test set, revealed no significant differences for LNM, FIGO III-IV or high-risk histology prediction (*p* ≥ 0.07 for all) (Table 2.).

Comparing R_dss_ with the specifically trained R_lnm_, R_figo_, and R_hist_ models (Online Resources 4, 5) in the test set, showed no significant differences in prediction accuracies for LNM, FIGO III-IV or high-risk histology (*p*** ≥ **0.15) (Table 4.).

**Table 4.** **Radiomic- and combined models for prediction of lymph node metastases, advanced stage and high-risk histology**. Area under receiver operating characteristic curves (AUC ROCs) for the radiomic disease-specific survival (DSS) score (R_dss_), the combined DSS score (C_dss_), and the specifically developed radiomic scores for prediction of lymph node metastases (R_lnm_), advanced FIGO III-IV stage (R_figo_) and high-risk histology (R_hist_). Pairwise AUC comparisons for C_dss_ versus R_dss_, R_lnm_, R_figo_ and R_his__t_ (p^b^), and for R_dss_ versus R_lnm_, R_figo_ and R_hist_ (p^c^) are given. All analyses are performed both in the training- (n = 343) and test (n = 146) sets

**Lymph node metastases**^**a**^** (AUC ROC)**	**C**_**dss**_^**e**^	**R**_**dss**_	**R**_**lnm**_
training (*p*^b^/*p*^c^), *n* = 211	.820 (-/-)	.686 (**/-)	.790 (ns/*)
test (*p*^b^/*p*^c^), *n* = 81	.800 (-/-)	.636 (ns/-)	.584 (*/ns)
**FIGO III-IV (AUC ROC)**	**C**_**dss**_^**e**^	**R**_**dss**_	**R**_**figo**_
training (*p*^b^/*p*^c^), *n* = 343	.857 (-/-)	.740 (**/-)	.762 (**/ns)
test (*p*^b^/*p*^c^), *n* = 146	.805 (-/-)	.739 (ns/-)	.745 (ns/ns)
**High-risk histology**^**d**^** (AUC ROC)**	**C**_**dss**_^**e**^	**R**_**dss**_	**R**_**hist**_
training (*p*^b^/*p*^c^), *n* = 338	.886 (-/-)	.612 (**/-)	.709 (**/**)
test (*p*^b^/*p*^c^), *n* = 146	.869 (-/-)	.686 (**/-)	.646 (**/ns)

*FIGO* International Federation of Gynecology and Obstetrics

^a^Lymphadenectomy in *n* = 292 patients

^b^*p*-value (deLongs test) for pairwise area under curve comparisons between C_dss_ versus R_dss_, R_lnm_, R_figo_, and R_hist_. deLong test *p* < 0.05 denoted (*), *p* < 0.01 denoted (**), *p* ≥ 0.05 denoted ns (not significant)

^c^*p*-value (deLongs test) for pairwise area under curve comparisons between R_dss_ versus R_lnm_, R_figo_, and R_hist_. deLong test *p* < 0.05 denoted (*), *p* < 0.01 denoted (**), *p* ≥ 0.05 denoted ns (not significant)

^d^Endometrioid grade 3 or non-endometrioid endometrial carcinoma. Grade missing for five patients

^e^Preoperative histology missing for eight patients. AUCs of C_dss_ and p-value comparisons with C_dss_ are therefore reported for in total *n* = 481 patients for FIGO, *n* = 207 patients for lymph node metastases, *n* = 477 patients for high-risk histology

The combined score C_dss_ yielded ROC AUCs of 0.820/0.800 for LNM, 0.857/0.805 for FIGO III-IV, and 0.886/0.869 for high-risk histology in the training/test sets (Table 2.). In the training set, C_dss_ yielded superior prediction of LNM, FIGO III-IV and high-risk histology compared to R_dss_ (*p* ≤ 0.007 for all). In the test set, this improved prediction was only observed for high-risk histology (*p* < 0.001) (Table 2.).

Comparing C_dss_ with the specifically trained R_lnm_, R_figo_, and R_hist_ models (Online Resources 4, 5) in the test set, showed significantly higher C_dss_ prediction accuracies for LNM (C_dss_/R_lnm_: 0.800/0.583, *p* = 0.03), and high-risk histology (C_dss_/R_hist_: 0.869/0.651, *p* < 0.001), although similar prediction accuracies for FIGO III-IV (C_dss_/R_figo_: 0.805/0.747, *p* = 0.26) (Table 4.).

## Discussion

In this study, we derived and evaluated primary tumor [^18^F]FDG PET radiomics for pretreatment prediction of aggressive disease and patient survival in a large endometrial cancer cohort. For prediction of survival, we constructed a radiomic model (R_dss_) and also a combined model (C_dss_), that included R_dss_, the conventional PET imaging finding LN_PET,_ and preoperative histology risk. The models were trained in a set of 343 endometrial cancer patients, and subsequently tested in a separate set of 146 patients.

In the test set, R_dss_ outperformed the conventional primary tumor markers SUV_max_ and SUV_mean_ for predicting 2- and 5-year DSS, but yielded similar accuracies to those of MTV, TLG, and LN_PET_. The prediction performance of R_dss_ declined from high 2-year DSS accuracy (AUC: 0.86) to lower 3- to 5-year DSS accuracies (AUC: 0.80–0.72). Interestingly, the combined model C_dss_ demonstrated excellent accuracy for predicting 2-year DSS (AUC: 0.91) and maintained high DSS accuracies at later time points (3- to 5-year AUCs: 0.87–0.82).

For predicting LNM, FIGO III-IV and high-risk histology in the test set, the prediction accuracies for R_dss_, SUV_max_, SUV_mean_, MTV, and TLG were generally moderate-to-low (AUC ≤ 0.74 for all). The routinely reported LN_PET_, showed moderate-to-high prediction accuracy for LNM (AUC: 0.82), and FIGO III-IV (AUC: 0.77), while low prediction accuracy for high-risk histology (AUC: 0.59). Conversely, the combined model C_dss_ exhibited high prediction accuracies for both LNM (AUC: 0.80), FIGO III-IV (AUC: 0.81) and high-risk histology (AUC: 0.87).

[^18^F]FDG PET radiomics have only to a limited extent been investigated in endometrial cancer, and to the best of our knowledge, this study is the largest and most comprehensive PET radiomic study to date. Previous studies [23–25] are based on smaller patients cohorts (ranging from 53 to 167 patients), reporting metrics of single/specific radiomic features for prediction of lymph node metastases [23, 24], and survival [25]. In a study including 53 patients, Nakajo et al., investigated 40 PET-based radiomic features for prediction of progression-free survival (PFS) and overall survival (OS) [25]. In multivariable analyses, the authors found the radiomic feature *Coarseness (NGTDM)* to be the only feature independently predicting PFS and OS. However, as noted by the authors, this study was limited by a small patient cohort inducing risk of patient selection bias, and lack of a training-test scheme to validate their findings.

The radiomic primary tumor features extracted in the present study, were computed according to the IBSI standard and all features were harmonized using the ComBat methodology to reduce scanner/protocol effects [30, 34, 35]. The R_dss_ model incorporated two morphological features (*SurfaceArea* and *Maximum3Ddiameter)*, one intensity histogram feature (*MaxiumHistogramGradient*), and one GLCM feature (*NormalisedInverseDifference*). After ComBat harmonization, no differences in distribution were observed for these features between the two scanners used. The R_dss_ survival prediction performance was moderate-to-high both in the training set (2- to 5-year DSS AUC: 0.77–0.64) and the test set (2- to 5-year DSS AUC: 0.86–0.72), indicating a high model generalizability.

For both R_dss_ and several of the conventional PET imaging markers in this study, we observed a decline in survival prediction accuracy over time. Similar declines in prediction accuracies have been reported for preoperative MRI radiomic features in an endometrial cancer study by Hodneland et al. [36]. Taken together, these findings suggest that while preoperative imaging markers and radiomics are highly predictive of endometrial cancer patient outcomes in the short term, their predictive power decreases over time. Interestingly, the combined model C_dss_, developed in the present study, demonstrated high survival prediction performance both in the short and long term. Additionally, the difference in survival rates between high- and low-C_dss_ scores was highly significant, and C_dss_ demonstrated more distinct risk stratification between the model score groups than R_dss_.

In addition to predicting survival, we also investigated PET radiomics for predicting LNM, FIGO III-IV and high-risk histology. In the test set, both R_dss_ and the specifically trained radiomic models (R_lnm_, R_figo_, R_hist_) yielded moderate-to-low prediction accuracies for LNM, FIGO III-IV, and high-risk histology, with AUCs ranging from 0.58 to 0.75. Conversely, the C_dss_ model yielded high prediction accuracies for all of the aggressive disease outcomes, with AUCs ranging from 0.80 to 0.87. For the prediction of LNM, De Bernardi et al. demonstrated in a study on 115 patients, that the radiomic PET feature *ZonePercentage (GLSZM)* outperformed both conventional and other radiomic features, achieving LNM sensitivity/specificity of 0.89/0.80 in a test set of 29 patients [24]. In a similar study on 167 patients, Crivellaro et al. identified the radiomic feature *VolumeDensity* (morphology/shape), as the most predictive of LNM, with sensitivity/specificity/AUC of 0.48/0.97/0.77 in a test set of 107 patients [23]. None of these studies addressed the protocol/scanner dependency of the PET radiomic features, which could potentially bias the results. The R_lnm_ model, specifically trained for LNM prediction in the present study, demonstrated high accuracy in the training set (AUC: 0.79). However, when applied to the test set, the R_lnm_ AUC dropped to 0.58, indicating substantial degree of model overfitting. This underscores the necessity of validating model and features in separate patient cohorts when conducting radiomic studies that incorporate a large number of imaging features.

The present study is to our knowledge the first to investigate PET radiomics for predicting advanced FIGO stage and histology type/grade in endometrial cancer. However, the prediction of stage and grade has been explored in a previous MRI radiomics study from our group, where whole-tumor radiomics were extracted in 138 endometrial cancer patients. Similar to R_dss_, R_figo_, and R_hist_, the predictive performances for the MRI radiomic models were only moderate for FIGO III-IV (AUC: 0.71/0.68) and high-risk histology (AUC NEEC: 0.68/0.74; AUC EEC grade 3: 0.79/0.63) in the training (*n* = 108)/test (*n* = 30) sets [19]. Combination models incorporating both MRI radiomics and other imaging/clinical findings were not explored in this previous study, but could potentially increase prediction performance for MRI radiomics, as observed for C_dss_ in the present study.

[^18^F]FDG PET/CT is considered the imaging modality of choice for preoperative assessment of disease spread in endometrial cancer. The routinely reported imaging marker LN_PET_ showed high diagnostic accuracy in the test set for predicting LNM (AUC: 0.82), FIGO III-IV (AUC: 0.77) and 2-year DSS (AUC: 0.80). Unlike the quantitative primary tumor markers (SUV_max/mean_, MTV and TLG), LN_PET_ is not reported as part of the radiomic/IBSI framework. Interestingly, when incorporating LN_PET_ into the combined model C_dss_ (together with R_dss_ and preoperative histology risk), the prediction accuracies were substantially increased for all the investigated outcomes, highlighting the importance of LN_PET_ assessments in endometrial cancer.

In addition to LN_PET_, primary tumor SUV_max_ is commonly included in clinical endometrial cancer imaging reports. SUV_max_ reflects a single voxel value and is therefore highly susceptible to variations in image reconstruction, and scanner/protocol settings. In this study, all features were harmonized to reduce scanner/protocol dependencies. Nevertheless, SUV_max_ demonstrated overall low test set accuracies for all the assessed outcomes (AUCs: 0.49–0.62) and was not significantly linked to DSS in univariable Cox analyses.

Although not commonly included in clinical endometrial cancer imaging reports, several previous studies have highlighted MTV and/or TLG as easily retrievable primary tumor markers with high diagnostic precision for preoperative staging and prediction of poor outcome in endometrial cancer [10, 21, 37, 38]. In the present study, MTV (and TLG) demonstrates similar prediction accuracies to those of R_dss_, and higher than those of SUV_max_ and SUV_mean_, for all investigated outcomes. These findings support the capacity of ‘size-like’ features, such as MTV, as valuable imaging markers that can aid in preoperative prognostication in endometrial cancer. Interestingly, two of the radiomic features incorporated in R_dss_, are metabolic tumor ‘size-like’ features (*SurfaceArea* and *Maximum3DDiameter).* Moreover, while both R_dss_ and MTV were significantly linked to DSS in univariable Cox analyses, only R_dss_ remained a significant DSS predictor in the multivariable analyses. PET radiomic modeling thus appears to add value compared to MTV for predicting poor outcome in endometrial cancer. This is further supported by the superior prediction accuracies of the combined C_dss_ model over MTV in this study.

### Limitations

Radiomic analyses, particularly the calculation of matrix-based texture features (GLCM, GLRLM, NGTDM, GLSZM), require MTVs to have minimum number of voxels. In the present study the threshold was set at 64 voxels. Consequently, 7% (34 out of 523) of the patients, whose MTVs were smaller than the threshold, were excluded. This inclusion criterion might theoretically skew the patient cohort towards more advanced disease. However, when comparing the present study cohort with the Norwegian endometrial cancer patient population, the proportion of patients with low stage disease (FIGO I: 74%) corresponds well with nationally reported cancer staging numbers (71% with localized disease [33]).

Before radiomic feature extraction, we investigated the tumor SUV intensity range in the entire patient population. Based on this, the minimum/maximum intensity bound were set to 0/40 SUV, and the intensity discretization to 128 grey levels, corresponding to a bin size of 0.32 SUV. There is no consensus on the optimal bin number/size for radiomic feature extraction, as it is likely problem/task/disease dependent [39]. In endometrial cancer, future studies are needed to determine the optimal radiomic feature extraction settings (such as bin size/bin width) to improve the diagnostic precision of PET radiomics.

In the present study, MTVs were defined using a 2.5 SUV threshold within a manually delineated volume of interest. The choice of threshold was based on previous [^18^F]FDG PET endometrial cancer studies, which demonstrated high prognostic value for MTV defined by a 2.5 SUV threshold [10, 21]. During the pilot phase of this project, we also investigated SUV_max_-based thresholds of 40–60% and observed that these yielded smaller MTVs compared to the 2.5 SUV threshold. Consequently, using these thresholds would result in higher patient exclusion rates due to the minimum voxels requirement for radiomic feature extraction (64 voxels). Additionally, the interreader agreement for MTV using the 2.5 SUV threshold was excellent (ICC: 0.97), and only 10 out of 108 radiomic features were excluded from radiomic modelling due to lower (≤ 0.75) ICC values. These findings suggest that deriving threshold-based MTVs, provides robust and reproducible radiomic features in endometrial cancer. However, more automated and less time-consuming MTV segmentation methods would enhance the feasibility of extracting MTV and/or radiomic profiling in clinical practice.

Currently, machine learning-based PET tumor segmentations, using state-of-the-art methods such as the U-Net CNN architecture, are being developed and will hopefully help automate tumor volume of interest definitions in future clinical PET software systems [34, 40]. However, in endometrial cancer, separating bladder signal from tumor signal is challenging even for human experts, and any automated method would need to be specifically trained in this task.

To account for interreader variability in tumor segmentation, only radiomic features with high ICCs should ideally be retained for radiomic prediction modelling. In this study, we selected an ICC threshold of 0.75 as the feature retention criterion. However, there is no established consensus on the ICC threshold for radiomic studies, and the definition of'excellent interreader agreement ranges from ICCs > 0.75 [41] to > 0.90 [42]. Nevertheless, most of the radiomic features incorporated in R_dss_, R_figo_, R_lnm_, and R_hist_ exhibited ICCs > 0.90 (Online Resource 5) and are thus minimally affected by reader variability in tumor delineation.

Various methods can be employed to develop radiomic models, including regression techniques, random forest trees, support vector machines, and more recently, machine learning and deep learning approaches. In this study, we utilized LASSO and LASSO Cox modeling primarily due to their capabilities in feature reduction/selection and the handling time-dependent survival as an output. For collinear variables, LASSO tends to select one variable from the group and shrink the other to zero. This aids in reducing multicollinearity since LASSO eliminates redundant variables that provide little additional information. However, recent studies suggest that ensemble models (combining multiple, diverse models) may enhance model performance [43–45], which would also be a promising avenue for future research in endometrial cancer.

Given that LASSO feature selection can be influenced by scale differences across radiomic features, we conducted LASSO modeling using both z-normalized features and non-normalized (original scale) features. As this had no effect on the resulting feature selection, penalizing parameter (lambda), or predictive model performance, we opted to retain the features in their original scale. This approach also facilitates easier application and testing of the models/coefficients derived in this study in other radiomic datasets.

### Conclusion

The [^18^F]FDG PET primary tumor radiomic score R_dss_ demonstrated high accuracy for predicting disease-specific survival in the short term (two years after primary treatment), but decreasing accuracy over time (three to five years after primary treatment). Compared to conventional imaging findings, R_dss_ outperformed tumor SUV_max/mean_ but showed similar survival prediction accuracy to MTV, TLG and LN_PET_. For predicting LNM, advanced FIGO stage and high-risk histology, the accuracies for R_dss_ were generally moderate-to-low.

Conversely, a combined model including R_dss_, LN_PET_ and preoperative histology risk demonstrated high-to-excellent accuracy for predicting both short- and long-term survival. The combined model also showed high accuracy for predicting LNM, advanced FIGO stage and high-risk histology.

In conclusion, combining PET primary tumor radiomics with the routinely reported preoperative markers (LN_PET_ and histology risk) may refine preoperative risk assessments, aid in tailoring treatment and predicting patient outcomes in endometrial cancer.

## Supplementary Information

Below is the link to the electronic supplementary material.

Supplementary file2 (DOCX 399 KB)

Supplementary file1 (XLSX 29 KB)

## Data Availability

The data sets generated and analyzed during the current study are not publicly available but are available from the corresponding author upon reasonable request, and if in compliance with the general data protection regulation (GDPR) and patient consents.
