# Supplementary material for: Predicting aggressive disease and poor outcome in endometrial cancer using preoperative [18F]FDG PET primary tumor radiomics
Source: Eur J Nucl Med Mol Imaging. 2025 Jun 11;53(1):167–80. doi: 10.1007/s00259-025-07335-7 (PMC12660434; doi:10.1007/s00259-025-07335-7)
Supplement: Supplementary file 1 — Supplementary file2 (DOCX 399 KB) [file 259_2025_7335_MOESM1_ESM.docx]

# Online/supplementary material

## Online Resource 1: Scanner settings

| [^18^F]FDG PET/CT acquisition and reconstruction parameters for the two scanners used: Siemens Truepoint 40 during 2011-2018 (n=348) and Siemens Biograph Vision from 2018-2020 (n=141) | | | | | | |
| --- | --- | --- | --- | --- | --- | --- |
| **PET** | **Scan time/speed** | **Corrections** | **Iteration/subset** | **Filter** | **FOV (mm)** | **Voxel size (mm)** |
| Truepoint 40 | 3 min/bed | ATTN/SCAT | 4/8 | 5 mm gauss | 700 | 4.1 × 4.1 × 5.0 |
| Biograph Vision | 1.1 mm/s | ATTN/SCAT/PSF/TOF | 4/5 | All pass | 700 | 1.7 × 1.7 × 3.0 |
|  |  |  |  |  |  |  |
| **CT** | **CareDose^b^ (ref mAs)** | **CarekV^b^ (kV)** | **Recon** | **Filter/IR strength** | **FOV scan/recon (mm)** | **Voxel size (mm)** |
| Truepoint 40 | Yes (50/240)^a^ | No (120) | FB | B19f | 500/700 | 1.4 × 1.4 × 5.0 |
| Biograph Vision | Yes (25/210)^a^ | Yes (120) | IR | I30f/5 | 500/780 | 1.5 × 1.5 × 3.0 |
| ATTN, attenuation correction; CT, computed tomography; [^18^F]FDG PET, [^18^F]fluoro-deoxy-glucose positrom emission tomography; FB, filtered backprojection; FOV, field of view; IR, iterative reconstruction; PSF, point spread function correction; SCAT, scatter correction; TOF, time of flight  ^a^Reference mAs for low-dose/diagnostic CT protocols  ^b^Automatic tube current/voltage modulation system on Siemens CT scanners | | | | | | |

## Online Resource 2: List of radiomic features extracted

| List of 108 IBSI compliant radiomic features extracted from primary tumors on [^18^F]FDG PET in a large endometrial cancer cohort (n=489). Only features with intraclass correlation coefficients (ICCs)>0.75 were included for further analyses. The ICCs are based on subcohort analyses between two individual readers in 154 patients | |
| --- | --- |
| **Morphological features** | |
| *Included, ICC>0.75 (11 features)*  FeatureName(IBSI:ID)[unit] (*ICC*) | *Excluded, ICC≤0.75 (1 feature)*  FeatureName(IBSI:ID)[unit] (*ICC*) |
| Volume(IBSI:RNU0)[mm3] (*0.971*)  ApproximateVolume(IBSI:YEKZ)[mm3] (*0.971*)  SurfaceArea(IBSI:C0JK)[mm2] (*0.958*)  SurfaceToVolumeRatio(IBSI:2PR5)[mm] (*0.977*)  Compactness1(IBSI:SKGS)[] (*0.859*)  Compactness2(IBSI:BQWJ)[] (*0.861*)  SphericalDisproportion(IBSI:KRCK)[] (*0.892*)  Sphericity(IBSI:QCFX)[] (*0.860*)  Asphericity(IBSI:25C7)[] (*0.892*)  Max3DDiameter(IBSI:L0JK)[mm] (*0.904*)  IntegratedIntensity(IBSI:99N0)[Intensity] (*0.937*) | CentreOfMassShift(IBSI:KLMA)[mm] (*0.710*) |
|  |  |
| **Intensity features** | |
| *Included, ICC>0.75 (16 features)*  FeatureName(IBSI:ID)[unit] (*ICC*) | *Excluded, ICC≤0.75 (2 features)*  FeatureName(IBSI:ID)[unit] (*ICC*) |
| MeanInt(IBSI:Q4LE)[SUVbw] (*0.939*)  IntVariance(IBSI:ECT3)[SUVbw] (*0.894*)  IntSkewness(IBSI:KE2A)[SUVbw] (*0.904*)  IntKurtosis(IBSI:IPH6)[SUVbw] (*0.920*)  MedianInt(IBSI:Y12H)[SUVbw] (*0.899*)  90thIntPercentile(IBSI:8DWT)[SUVbw] (*0.950*)  MaxInt(IBSI:84IY)[SUVbw] (*0.986*)  IntInterquartileRange(IBSI:SALO)[SUVbw] (*0.948*)  IntRange(IBSI:2OJQ)[SUVbw] (*0.986*)  IntBasedMeanAbsoluteDeviation(IBSI:4FUA)[SUVbw] (*0.954*)  IntBasedRobustMeanAbsoluteDeviation(IBSI:1128)[SUVbw] (*0.950*)  IntBasedMedianAbsoluteDeviation(IBSI:N72L)[SUVbw] (*0.954*)  IntBasedCoefficientOfVariation(IBSI:7TET)[SUVbw] (*0.952*)  IntBasedQuartileCoefficientOfDispersion(IBSI:9S40)[SUVbw] (*0.949*)  IntBasedEnergy(IBSI:N8CA)[SUVbw] (*0.997*)  RootMeanSquareInt(IBSI:5ZWQ)[SUVbw] (*0.949*) | MinInt(IBSI:1GSF)[SUVbw] (*0.065*)  10thIntPercentile(IBSI:QG58)[SUVbw] (*0.433*) |
|  |  |
| **Intensity histogram features** | |
| *Included, ICC>0.75 (19 features)*  FeatureName(IBSI:ID)[unit] (*ICC*) | *Excluded, ICC≤0.75 (4 features)*  FeatureName(IBSI:ID)[unit] (*ICC*) |
| IntHistMean(IBSI:X6K6)[Intensity] (*0.942*)  IntHistVariance(IBSI:CH89)[Intensity] (*0.918*)  IntHistSkewness(IBSI:88K1)[Intensity] (*0.899*)  IntHistKurtosis(IBSI:C3I7)[Intensity] (*0.918*)  IntHistMedian(IBSI:WIFQ)[Intensity] (*0.895*)  IntHist90thPercentile(IBSI:OZ0C)[] (*0.954*)  IntHistMaxGreyLevel(IBSI:3NCY)[Intensity] (*0.996*)  IntHistMode(IBSI:AMMC)[Intensity] (*0.806*)  IntHistInterquartileRange(IBSI:WR0O)[Intensity] (*0.946*)  IntHistRange(IBSI:5Z3W)[Intensity] (*0.996*)  IntHistMeanAbsoluteDeviation(IBSI:D2ZX)[Intensity] (*0.960*)  IntHistRobustMeanAbsoluteDeviation(IBSI:WRZB)[Intensity] (*0.926*)  IntHistMedianAbsoluteDeviation(IBSI:4RNL)[Intensity] (*0.959*)  IntHistCoefficientOfVariation(IBSI:CWYJ)[Intensity] (*0.956*)  IntHistQuartileCoefficientOfDispersion(IBSI:SLWD)[Intensity] (*0.943*)  IntHistEntropyLog2(IBSI:TLU2)[Intensity] (*0.982*)  Uniformity(IBSI:BJ5W)[Intensity] (*0.968*)  MaxHistGradient(IBSI:12CE)[Intensity] (*0.782*)  MaxHistGradientGreyLevel(IBSI:8E6O)[Intensity] (*0.810*) | IntHistMinGreyLevel(IBSI:1PR8)[Intensity] (*0.009*)  IntHist10thPercentile(IBSI:GPMT)[] (*0.466*)  MinHistGradient(IBSI:VQB3)[Intensity] (*0.644*)  MinHistGradientGreyLevel(IBSI:RHQZ)[Intensity] (*0.035*) |
| [^18^F]FDG PET, [^18^F]fluorodeoxyglucose positrom emission tomography; IBSI, image biomarker standardisation initiative | |
| *Cont. Online Resource 2* |  |
| **Grey-Level Co-occurremce Matrix (GLCM)** | |
| *Features included, ICC>0.75 (21 features)*  FeatureName(IBSI:ID) (*ICC*) | *Features excluded, ICC≤0.75 (2 features)*  FeatureName(IBSI:ID)[unit] (*ICC*) |
| JointMax(IBSI:GYBY) (*0.878*)  JointAverage(IBSI:60VM) (*0.948*)  JointVariance(IBSI:UR99) (*0.930*)  JointEntropyLog2(IBSI:TU9B) (*0.986*)  DifferenceAverage(IBSI:TF7R) (*0.921*)  DifferenceEntropy(IBSI:NTRS) (*0.986*)  SumAverage(IBSI:ZGXS) (*0.948*)  SumVariance(IBSI:OEEB) (*0.962*)  SumEntropy(IBSI:P6QZ) (*0.995*)  AngularSecondMoment(IBSI:8ZQL) (*0.977*)  Dissimilarity(IBSI:8S9J) (*0.921*)  InverseDifference(IBSI:IB1Z) (*0.971*)  NormalisedInverseDifference(IBSI:NDRX) (*0.966*)  InverseDifferenceMoment(IBSI:WF0Z) (*0.966*)  NormalisedInverseDifferenceMoment(IBSI:1QCO) (*0.967*)  InverseVariance(IBSI:E8JP) (*0.986*)  Correlation(IBSI:NI2N) (*0.982*)  Autocorrelation(IBSI:QWB0) (*0.937*)  ClusterTendency(IBSI:DG8W) (*0.962*)  ClusterShade(IBSI:7NFM) (*0.924*)  ClusterProminence(IBSI:AE86) (*0.932*) | DifferenceVariance(IBSI:D3YU) (*0.731*)  Contrast(IBSI:ACUI) (*0.713*) |
|  |  |
| **Grey-Level Run-Length Matrix (GLRLM)** | |
| *Features included, ICC>0.75 (11 features)*  FeatureName(IBSI:ID)[unit] (*ICC*) | *Features excluded, ICC≤0.75 (0 features)*  FeatureName(IBSI:ID)[unit] (*ICC*) |
| ShortRunsEmphasis(IBSI:22OV) (*0.942*)  LongRunsEmphasis(IBSI:W4KF) (*0.933*)  LowGreyLevelRunEmphasis(IBSI:V3SW) (*0.917*)  HighGreyLevelRunEmphasis(IBSI:G3QZ) (*0.921*)  ShortRunLowGreyLevelEmphasis(IBSI:HTZT) (*0.920*)  ShortRunHighGreyLevelEmphasis(IBSI:GD3A) (*0.924*)  LongRunLowGreyLevelEmphasis(IBSI:IVPO) (*0.900*)  LongRunHighGreyLevelEmphasis(IBSI:3KUM) (*0.937*)  GreyLevelNonUniformity(IBSI:R5YN) (*0.960*)  RunLengthNonUniformity(IBSI:W92Y) (*0.985*)  RunPercentage(IBSI:9ZK5) (*0.934*) | n/a |
|  |  |
| **Neighbouring Gray Tone Difference Matrix (NGTDM)** | |
| *Features included, ICC>0.75 (4 features)*  FeatureName(IBSI:ID)[unit] (*ICC*) | *Features excluded, ICC≤0.75 (1 feature)*  FeatureName(IBSI:ID)[unit] (*ICC*) |
| Coarseness(IBSI:QCDE) (*0.929*)  Contrast(IBSI:65HE) (*0.770*)  Busyness(IBSI:NQ30) (*0.965*)  Strength(IBSI:1X9X) (*0.969*) | Complexity(IBSI:HDEZ) (*0.658*) |
|  |  |
| **Grey-Level Size Zone Matrix (GLSZM)** | |
| *Features included, ICC>0.75 (16 features)*  FeatureName(IBSI:ID)[unit] (*ICC*) | *Features excluded, ICC≤0.75 (0 feature)*  FeatureName(IBSI:ID)[unit ] (*ICC*) |
| SmallZoneEmphasis(IBSI:5QRC) (*0.964*)  LargeZoneEmphasis(IBSI:48P8) (*0.959*)  LowGrayLevelZoneEmphasis(IBSI:XMSY) (*0.972*)  HighGrayLevelZoneEmphasis(IBSI:5GN9) (*0.966*)  SmallZoneLowGreyLevelEmphasis(IBSI:5RAI) (*0.923*)  SmallZoneHighGreyLevelEmphasis(IBSI:HW1V) (*0.955*)  LargeZoneLowGreyLevelEmphasis(IBSI:YH51) (*0.897*)  LargeZoneHighGreyLevelEmphasis(IBSI:J17V) (*0.985*)  GreyLevelNonUniformity(IBSI:JNSA) (*0.987*)  NormalisedGreyLevelNonUniformity(IBSI:Y1RO) (*0.982*)  ZoneSizeNonUniformity(IBSI:4JP3) (*0.995*)  NormalisedZoneSizeNonUniformity(IBSI:VB3A) (*0.957*)  ZonePercentage(IBSI:P30P) (*0.943*)  GreyLevelVariance(IBSI:BYLV) (*0.955*)  ZoneSizeVariance(IBSI:3NSA) (*0.961*)  ZoneSizeEntropy(IBSI:GU8N) (*0.992*) | n/a |
| [^18^F]FDG PET, [^18^F]fluorodeoxyglucose positrom emission tomography; IBSI, image biomarker standardisation initiative | |

## Online Resource 3: Radiomic features before and after ComBat harmonization (see separate data sheet)

## Online Resource 4: LASSO modelling calibration plots

Radiomic models predicting disease specific survival (DSS) (**a, e**), lymph node metastases (LNM) (**b, f**), FIGO III-IV stage (**c, g**) and high-risk histology (**d, h**) were developed using least absolute shrinkage and selection operator (LASSO) Cox or LASSO regression. Optimal lambda (λ, dotted line) was selected by ten-fold cross-validation and minimizing the partial likelihood/binominal deviance (**a-d**), and coefficient profiles for different values of λ were given for each of the models in (**e-h**)


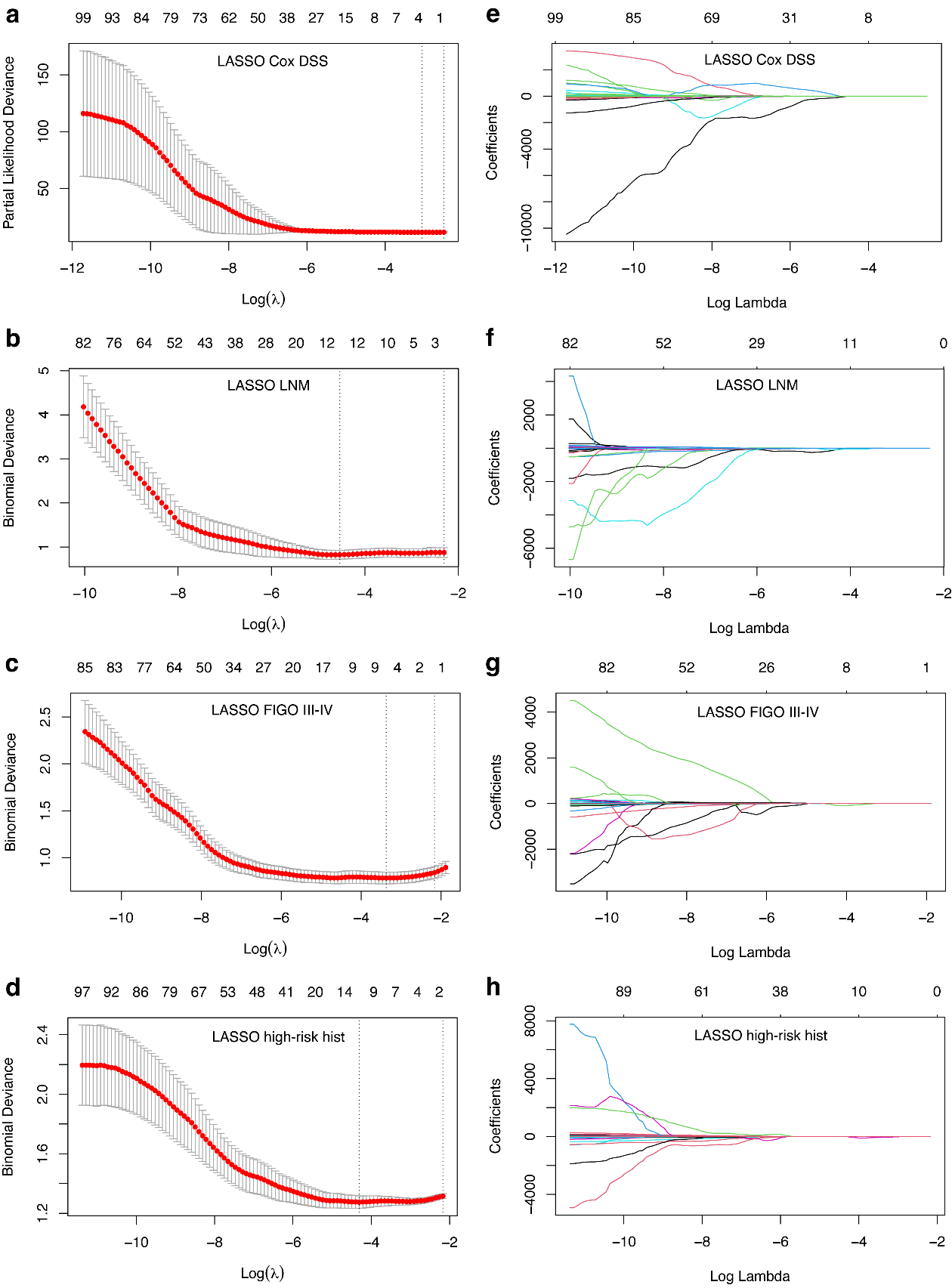


## Online Resource 5: Radiomics models for aggressive disease

| Radiomic least absolute shrinkage and selection operator (LASSO) Cox model for prediction of disease specific survival (R_dss_), and LASSO models for prediction of surgically verified lymph node metastases (R_lnm_), FIGO 2009 stage III-IV (R_figo_) and high-risk histology^a^ (R_hist_) | | |
| --- | --- | --- |
| **Radiomic model** | **Radiomic model features selected by LASSO Cox/LASSO**  FeatureClass_FeatureName(IBSI:ID)[unit] (*ICC*) | **LASSO**  **coefficients** |
| **R_dss_** | Morph*_SurfaceArea(IBSI:C0JK)[mm2] (0.958)*  Morph*_Maximum3DDiameter(IBSI:L0JK)[mm] (0.904)*  IntHist*_MaximumHistGradient(IBSI:12CE)[Intensity] (0.782)*  GLCM*_NormalisedInverseDifference(IBSI:NDRX) (0.966)* | *.00001131625*  *.00093168718*  *.00098847583*  *.79025244362* |
|  |  |  |
| **R_lnm_** | Morph*_Maximum3DDiameter(IBSI:L0JK)[mm] (0.904)*  IntHist*_MaximumHistGradient(IBSI:12CE)[Intensity] (0.782)*  IntHist*_MaximumHistGradientGreyLevel(IBSI:8E6O)[Intensity] (0.810)*  GLCM_*JointMaximum(IBSI:GYBY) (0.878)*  GLCM_*SumVariance(IBSI:OEEB) (0.962)*  GLCM_*InverseVariance(IBSI:E8JP) (0.962)*  GLCM_*ClusterTendency(IBSI:DG8W) (0.962)*  GLRLM_*LongRunHighGreyLevelEmphasis(IBSI:3KUM) (0.937)*  NGTDM_*Busyness(IBSI:NQ30) (0.965)*  GLSZM_*SmallZoneEmphasis(IBSI:5QRC) (0.964)*  GLSZM_*SmallZoneLowGreyLevelEmphasis(IBSI:5RAI) (0.923)*  GLSZM*_LargeZoneLowGreyLevelEmphasis(IBSI:YH51) (0.897)*  *(Intercept)* | *.00318856146*  *.00464717897*  *-.00338048333*  *-7.1959142739*  *.00051822735*  *-201.41345069*  *.00007813294*  *.00047393475*  *-.14378862820*  *-7.8536101016*  *-81.774045063*  *-.09695296905*  *(2.6960607874)* |
|  |  |  |
| **R_figo_** | Morph*_SurfaceArea(IBSI:C0JK)[mm2] (0.958)*  Morph*_Maximum3DDiameter(IBSI:L0JK)[mm] (0.904)*  Int_*MedianIntensity(IBSI:Y12H)[SUVbw] (0.899)*  IntHist_*IntensityHistMedian(IBSI:WIFQ)[Intensity] (0.895)*  GLRLM_*LongRunHighGreyLevelEmphasis(IBSI:3KUM) (0.937)*  GLSZM_*SmallZoneLowGreyLevelEmphasis(IBSI:5RAI) (0.923)*  GLSZM_*ZoneSizeNonUniformity(IBSI:4JP3) (0.995)*  *(Intercept)* | *.00001090431*  *.01256594930*  *.03392642959*  *.00242802134*  *.00000174959*  *-10.692784687*  *.00078189868*  *(-2.9585650082)* |
|  |  |  |
| **R_hist_** | Morph*_SurfaceArea(IBSI:C0JK)[mm2] (0.958)*  Morph_*Compactness2(IBSI:BQWJ)[] (0.861)*  Morph*_Maximum3DDiameter(IBSI:L0JK)[mm] (0.904)*  Int_*IntensityBasedEnergy(IBSI:N8CA)[SUVbw] (0.997)*  IntHist_*IntensityHistSkewness(IBSI:88K1)[Intensity] (0.899)*  IntHist­_*MaximumHistGradientGreyLevel(IBSI:8E6O)[Intensity] (0.810)*  GLCM_*JointMaximum(IBSI:GYBY) (0.878)*  GLCM_*ClusterProminence(IBSI:AE86) (0.932)*  GLRLM_*ShortRunLowGreyLevelEmphasis(IBSI:HTZT) (0.920)*  NGTDM_*Busyness(IBSI:NQ30) (0.965)*  GLSZM_*LargeZoneHighGreyLevelEmphasis(IBSI:J17V) (0.985)*  *(Intercept)* | *.00006402219*  *2.3866136948*  *.00958187304*  *-.00000004146*  *-.00284538820*  *.02125940976*  *-3.5367244394*  *.00000000491*  *-14.663194263*  *-.46871142392*  *.00000479021*  *(-2.4221870069)* |
| FIGO, International Federation of Gynecology and Obstetrics; GLCM, grey-level co-occurrence matrix; GLRLM, grey-level run-length matrix; NGTDM, neighbouring gray tone difference matrix; GLSZM, grey-level size zone matrix; IBSI, image biomarker standardisation initiative  ^a^Endometrioid grade 3 or non-endometrioid endometrial carcinoma | | |
